# Correlation Analysis of Gut Microbiota Derivatives with Coronary Artery Disease Severity and Prognosis

**DOI:** 10.31083/RCM26566

**Published:** 2025-04-18

**Authors:** Yifeng Bai, Chunrong Jin, Hui Zhang, Yuanyang Jia, Shan Xiao, Yongjiang Yang

**Affiliations:** ^1^The First Clinical Medical College, Shanxi Medical University, 030000 Taiyuan, Shanxi, China; ^2^Department of Cardiology, First Hospital of Shanxi Medical University, 030000 Taiyuan, Shanxi, China

**Keywords:** coronary artery disease, derivatives of gut microbiota, Gensini score, coronary flow reserve, prognosis

## Abstract

**Background:**

Previous research has highlighted a connection between gut microbiota derivatives and atherosclerosis. This study assesses the association between gut microbiota derivatives and coronary artery disease (CAD) to enhance CAD prevention and treatment strategies.

**Methods:**

Patients presenting with suspected CAD were categorized into CAD and non-CAD groups. A propensity score matching analysis was performed to exclude confounding factors. Key differences in general characteristics and gut microbiota derivatives between these groups were also assessed. Additionally, the study explored the correlation between significant differences in the Gensini score and coronary flow reserve. Moreover, the potential of significant indicators to predict the diagnosis of coronary artery disease was analyzed.

**Results:**

After propensity score matching, the concentrations of interleukin-6 (IL-6) (47.23 ± 7.45 vs. 39.56 ± 7.37; *p* < 0.001), lipopolysaccharide (LPS) (12.79 ± 2.07 vs. 11.71 ± 1.88; *p* = 0.031), high-sensitivity C-reactive protein (hs-CRP) (13.58 ± 2.62 vs. 11.57 ± 2.49; *p* = 0.002), phenylacetyl glutamine (PAGIn) (619.20 ± 119.33 vs. 555.64 ± 109.29; *p* = 0.029), and trimethylamine-N-oxide (TMAO) (13.01 ± 2.19 vs. 11.70 ± 1.78; *p* = 0.011) in the CAD group were significantly elevated compared to those in the non-CAD group. Conversely, the serum levels of glucagon-like peptide-1 (GLP-1) (7.74 ± 2.07 vs. 9.06 ± 2.11; *p* = 0.012) were notably lower in the CAD group than in the non-CAD group. A positive association was observed between the serum concentrations of IL-6 (r = 0.410; *p* < 0.001), hs-CRP (r = 0.317; *p* < 0.007), TMAO (r = 0.311; *p* < 0.008), and coronary Gensini score. Moreover, IL-6 (b = 1.769, 95% confidence interval (CI): 0.256–3.282; *p* = 0.023) and TMAO (b = 10.735, 95% CI: 4.883–16.588; *p* < 0.001) had a direct positive impact on the coronary Gensini score. The highest diagnostic value for CAD was observed when the IL-6 cut-off value was 45.17 (sensitivity 69.6%, specificity 73.1%, area under curve 0.770; 95% CI: 0.662–0.879; *p* < 0.001). Meanwhile, the highest diagnostic value for CAD was noted when the TMAO cut-off value was 12.44 (sensitivity 65.2%, specificity 76.9%, the area under the curve 0.689; 95% CI: 0.564–0.814; *p* = 0.008). Serum TMAO was negatively correlated with coronary flow reserve (CFR) in CAD patients (r = –0.593; *p* = 0.009).

**Conclusions:**

These findings suggest that serum IL-6, LPS, hs-CRP, PAGIn, TMAO, and GLP-1 levels can be used as clinical markers for predicting CAD severity. Among these, IL-6, hs-CRP, and TMAO are identified as independent risk factors influencing the severity of CAD—elevated levels of IL-6 and TMAO exhibit predictive utility for CAD diagnosis. Furthermore, serum TMAO is a potential clinical marker for forecasting a CAD prognosis.

## 1. Introduction

Atherosclerosis (AS) serves as a major contributing factor to coronary artery disease (CAD), stroke, and peripheral artery disease [1]. Despite notable advancements in preventive measures, pharmacological treatment, and interventional procedures, the incidence and mortality rates of coronary artery disease continue to rise annually [2]. Detecting AS at an early stage and initiating timely intervention are recognized as effective strategies for both its prevention and treatment.

Recent research underscores the pivotal role of the gut microbiota in modulating a wide range of metabolic processes within the human body [3]. Specifically, the byproducts of gut microbiota activity, including metabolites such as lipopolysaccharide (LPS), short chain fatty acids (SCFA), trimethylamine-N-oxide (TMAO), total bile acid (TBA), phenylacetyl glutamine (PAGIn), tryptophan (TRP), interleukin-6 (IL-6), and high-sensitivity C-reactive protein (hs-CRP), have been implicated in the exacerbation of systemic inflammation. These compounds have been shown to influence key pathological processes such as foam cell accumulation, endothelial dysfunction, and lipid accumulation [4, 5]. These factors can also contribute to the development of AS, although the underlying mechanisms are multifactorial. Ongoing investigations into the relationship between gut microbiota derivatives and AS are essential for advancing prevention and therapeutic strategies of AS [6].

In clinical practice, coronary arteriography (CAG) and coronary computed tomography angiography (CTA) are the standard imaging modalities employed to diagnose CAD and assess its severity. For evaluating the extent of coronary lesions and overall disease burden, scoring systems such as Synergy between Percutaneous Coronary Intervention with Taxus and Cardiac Surgery (SYNTAX) and Gensiniare commonly utilized. Another important diagnostic concept is coronary flow reserve (CFR), a parameter that quantifies the ability of coronary vessels to dilate in response to stress. Introduced by Gould *et al*. in 1974 [7], CFR is calculated as the ratio of coronary blood flow (CBF) or myocardial blood flow (MBF) during induced stress to the baseline CBF or MBF [8].

A growing body of evidence has established that a reduced CFR is strongly associated with adverse clinical outcomes in CAD patients, including increased all-cause mortality and a higher incidence of major adverse cardiac events (MACE) [9]. Nevertheless, these prognosis methods are invasive and associated with significant financial costs. Hence, the identification of novel, minimally invasive indicators for accurately predicting the prognosis of AS and CAD holds substantial clinical importance.

## 2. Methods

### 2.1 Selection of Patients

This study enrolled 91 individuals suspected of having CAD who were admitted to the Department of Cardiology at the First Hospital of Shanxi Medical University between January 2020 to May 2022. The study was obtained permission from the Ethics Committee of First Hospital of Shanxi Medical University (Ethics approval number: [2019] K-SK037). Consent for data use has been consented by the patients. All participants subsequently underwent CAG as part of their diagnostic evaluation. The cohort included 59 males with a mean age of 59.37 ± 9.43 years and 32 females with a mean age of 59.54 ± 9.50 years. The severity of CAD in each patient was quantified using the Gensini score, while their prognosis was assessed by measuring CFR.

Inclusion criteria for this study were as follows: (1) age between 18 and 75 years; (2) admission to the Department of Cardiology at the First Hospital of Shanxi Medical University from January 2020 to May 2022 due to CAD, with subsequent CAG; (3) availability of complete clinical data.

Exclusion criteria included: (1) coexistence of congenital heart disease or heart valve disease; (2) previous interventions, such as percutaneous coronary intervention (PCI) or coronary artery bypass graft (CABG); (3) presence of severe arrhythmias such as atrioventricular block above degree II or sick sinus syndrome; (4) systolic blood pressure below 90 mmHg or heart rate lower than 40 beats per minute; (5) presence of asthma or diabetes; (6) concurrent severe infection, organ injury/failure, malignant tumor, or other diseases; (7) presence of any physical/mental conditions that hindered cooperation during an examination.

### 2.2 Data Collection

Upon the patient’s admission to the hospital, an initial assessment was conducted, which included the collection of essential demographic and clinical data. This encompassed information such as the patient’s sex, age, height, weight, body mass index (BMI), along with relevant medical history, including smoking habits and the presence of hypertension.

Blood samples were collected the following morning after admission, ensuring the patients were in a fasting state. The samples were then subjected to centrifuged at 3000 r/min for 15 minutes to separate the components. The resulting supernatant was stored at –80 °C to maintain the integrity of the samples for future analysis. For the quantification of IL-6, LPS, hs-CRP, PAGIn, TMAO, TBA, and glucagons-like peptide-1 (GLP-1), an ELISA kit (Quanzhou Ruixin Biological Technology Co.,LTD, Quanzhou, China) was employed. To ensure accurate measurement, a standard curve was established by correlating optical density (OD) with the known concentrations of the standards. The OD value obtained for each sample was then applied to the linear regression equation derived from this curve. The assay was repeated twice, and the average concentration from the two independent runs was used as the final value for each sample.

### 2.3 Coronary Arteriography

The coronary arteriography procedure was conducted through the radial artery. A 6F sheath was inserted, guided by a guide wire, to position the arteriography catheter at the ostia of the left and right coronary arteries. To ensure comprehensive imaging, multiple viewing angles were utilized. These included the following combinations of positions: left anterior oblique at 45 degrees with a 30-degrees footward tilt; left anterior oblique at 45 degrees with a 30-degrees headward tilt; right anterior oblique at 30 degrees with a 30-degrees headward tilt; right anterior oblique at 30 degrees with a 30-degrees footward tilt. For assessment of the right coronary artery, two separate positions were used, with both positioned at a left anterior oblique angle of 45 degrees and a headward tilt of 30 degree. The interpretation of the coronary angiography results was carried out by minimum of two experienced interventional cardiologists to ensure accuracy and reliability.

#### 2.3.1 Grouping

Based on coronary arteriography findings, the study population was categorized into two primary groups: the coronary artery disease group and the non-coronary artery disease group. The criteria for classification were as follows:

Patients who exhibited stenosis of 50% or more in any of the major coronary vessels—such as the left main artery, left anterior descending artery, left circumflex artery, and right coronary artery-along with their key branches (including but not limited to the diagonal, septal, obtuse marginal, posterior left ventricular, posterior descending, and sharp marginal branch), were allocated to the coronary artery disease group (46 cases).

In contrast, those whose coronary arteries showed stenosis of less than 50% in the same vessels and branches were classified into the non-coronary artery disease group. The distinction between these groups was based solely on the degree of stenosis observed, with no additional clinical factors being considered in the classification (45 cases).

#### 2.3.2 Positron Emission Tomography (PET) Myocardial Perfusion Imaging

All subjects provided informed consent for drug stress myocardial perfusion imaging prior to the examination. The subjects were required to fast (except for drinking water) for a minimum of 4 hours before the examination. Theophylline, vasodilators, and other medications were prohibited within 36 hours prior to the examination. Tea, coffee, cola, soda, and other caffeinated beverages containing theophylline were not allowed within 24 hours before the examination. Myocardial perfusion imaging (MPI) was conducted using PET. Prior to the examination, an indwelling needle was inserted on the back of the left hand of each subject. During the examination, patients were positioned in a supine position with raised hands and instructed to keep their head and chest still. Real-time monitoring of electrocardiogram (lead I, II, III), heart rate, and blood pressure from the right upper arm was performed throughout the procedure. After stabilization of the heart rate, a low-dose plain scan was conducted for attenuation correction and scanning range localization (between tracheal bifurcation and approximately 2 cm below apex). PET program initiation followed by injection of 13N-ammonia imaging agent (approximately 15mCi) enabled continuous dynamic data collection lasting around 12 minutes to complete resting positron emission tomography myocardial perfusion imaging (PET MPI). Adenosine injection at a total dose of 0.8 mg/KG with a flow rate of 0.140 mg/KG/min served as a loading drug during the stress phase. The blood pressure and heart rate measurements in were recorded starting from adenosine injection until approximately 15 minutes later. Patients underwent observation for thirty minutes after completion of the examination before being discharged. The GE Discovery VCT PET/CT (GE Healthcare, 3000 North Grandview Blvd, Waukesha, WI, USA) scanner with a capacity of sixty-four rows was utilized for this procedure.

Image processing: ACQC software (built into the system, GE Healthcare, 3000 North Grandview Blvd, Waukesha, WI, USA) was utilized for displacement correction reconstruction. The PET data collected during rest and load were divided into two phases, namely the blood pool phase of the first 2 minutes and the myocardial uptake phase of the subsequent 10 minutes, resulting in a total of four data groups. Software analysis and calculations were performed to assess rest and load MBF, CFR, as well as cardiac function parameters for both the entire ventricle and each individual wall.

#### 2.3.3 Gensini Score Calculation

The results of CAD was quantified using the Gensini scoring system, which is widely recognized for its ability to evaluate the extent and distribution of coronary artery stenosis [10]. In this system, coronary arteries are categorized into several key segments, including left main artery (LM), left anterior descending artery (LAD), left circumflex artery (LCX), and right coronary artery (RCA). Each of these arterial segments is assessed for the degree of stenosis, with a scoring scale that assigns points based on the percentage of luminal narrowing. Specifically, a stenosis of 25% or less corresponds to a score of 1, 26–50% stenosis is given 2 points, 51–75% stenosis is assigned 4 points, 76–90% stenosis earns 8 points, 91–99% stenosis is allocated 16 points, and total occlusion (100% stenosis) receives the highest score of 32 points. To account for the differing clinical significance of stenosis at various points along the coronary arteries, a weighted scoring system is applied. For instance, the left main artery, being a critical vessel, is assigned a multiplier of 5, reflecting its greater importance. Similarly, the proximal segment of the LAD is weighted by a factor of 2.5, while the middle portion of the LAD is assigned a multiplier of 1.5. The distal segment of the LAD and the first diagonal branch both have a weight of 1, with the second diagonal branch receiving a lower weight of 0.5. For the left circumflex artery, the proximal part is weighted at 2.5, and the distal and posterior descending branches are scored at 1 point each, with the posterior lateral branch given a weight of 0.5. Regarding the right coronary artery, its various segments—proximal, middle, distal, and posterior descending—are all assigned a score of 1. The final Gensini scores was determined by summing the weighted scores for all individual segment.

### 2.4 Statistical Methods

Data analysis was conducted using SPSS version 26.0 (IBM Corp., Chicago, IL, USA). To reduce the influence of selection bias and potential confounders, propensity score matching (PSM) was used to generate better matched groups. Descriptive statistics for normally distributed variables were presented as mean ± standard deviation ($\bar{x}\pm s$). To compare differences between groups for these variables, the independent sample *t*-test was applied. For variables that did not follow a normal distribution, date were expressed as the medians with interquartile ranges (M (P25, P75)), and group differences were assessed using the Wilcoxon rank-sum test. Categorical variables were summarized as percentages, and group comparisons were performed using either the Chi-square test or Fisher’s exact test, depending on the nature of the data. Spearman’s correlation analysis was employed to explore potential associations between risk factors and both the severity and prognosis of coronary artery disease. In addition, multivariate linear regression analysis was utilized to identify independent predictors of CAD severity. The diagnostic accuracy for CAD was assessed through the construction of a receiver operating characteristic (ROC) curve. A significance level of *p* < 0.05 was considered statistically meaningful for all tests.

## 3. Results

### 3.1 General Data Analysis of the CAD Group and Non-CAD Group

According to the results of Coronary arteriography, a total of 91 patients were categorized into the CAD group (46 cases, 50.5%) and non-CAD group (45 cases, 49.5%). After PSM, a total of 72 patients were categorized into the CAD group (46 cases, 63.9%) and non-CAD group (26 cases, 36.1%). Upon examining the general characteristics of both groups, no significant differences were observed in factors such as age, sex, height, weight, BMI, smoking habits or the prevalence of hypertension. For more detailed demographic data, refer to Table 1.

**Table 1.** **Comparison of general data between CAD group and non-CAD group**.

		Before PSM			After PSM		
		CAD group (n = 46)	Non-CAD group (n = 45)	*p* value	CAD group (n = 46)	Non-CAD group (n = 26)	*p* value
Age (y, $\bar{x}\pm s$)		60.19 ± 8.89	59.75 ± 11.13	0.893	60.19 ± 8.89	59.90 ± 11.49	0.992
Sex, n (%)				0.067			0.448
	Male	34 (73.9)	25 (55.6)		34 (73.9)	17 (65.4)	
	Female	12 (26.1)	20 (44.4)		12 (26.1)	9 (34.6)	
Height (m, $\bar{x}\pm s$)		1.66 ± 0.07	1.63 ± 0.07	0.223	1.66 ± 0.07	1.65 ± 0.10	0.679
Weight (kg, $\bar{x}\pm s$)		66.86 ± 10.03	65.63 ± 6.00	0.699	66.86 ± 10.03	65.60 ± 6.24	0.554
BMI (kg/m^2^, $\bar{x}\pm s$)		24.18 ± 3.09	24.79 ± 2.22	0.553	24.18 ± 3.09	24.49 ± 2.59	0.826
Smoking, n (%)		31 (67.4)	12 (26.7)	<0.001*	31 (67.4)	11 (42.3)	0.039
Hypertension, n (%)		23 (50.0)	19 (42.2)	0.457	23 (50.0)	12 (46.2)	0.967

CAD, coronary artery disease; PSM, propensity score matching; BMI, body mass index; *p < 0.05, the difference was statistically significant.

### 3.2 Analysis of the Serum Levels of Derivatives of Gut Microbiota in the CAD Group and Non-CAD Group

Serum analyses revealed that levels of IL-6 (47.23 ± 7.45 vs 39.56 ± 7.37, *p* < 0.001), LPS (12.79 ± 2.07 vs 11.71 ± 1.88, *p* = 0.031), hs-CRP (13.58 ± 2.62 vs 11.57 ± 2.49, *p* = 0.002), PAGIn (619.20 ± 119.33 vs 555.64 ± 109.29, *p* = 0.029) and TMAO (13.01 ± 2.19 vs 11.70 ± 1.78, *p* = 0.011) were considerably elevated in the CAD group compared to the non-CAD group. On the other hand, the CAD group (7.74 ± 2.07 vs 9.06 ± 2.11, *p* = 0.012) displayed a significantly reduced serum level of GLP-1 in comparison to the non-CAD group. These results are summarized in Table 2.

**Table 2.** **Comparison of the serum levels of derivatives of gut microbiota indexes between CAD group and non-CAD group**.

	Before PSM			After PSM		
	CAD group (n = 46)	non-CAD group (n = 45)	*p* value	CAD group (n = 46)	non-CAD group (n = 26)	*p* value
IL-6 (pg/mL, $\bar{x}\pm s$)	47.23 ± 7.45	41.81 ± 7.63	0.001*	47.23 ± 7.45	39.56 ± 7.37	<0.001*
LPS (pg/mL, $\bar{x}\pm s$)	12.79 ± 2.07	11.97 ± 1.84	0.049*	12.79 ± 2.07	11.71 ± 1.88	0.031*
hs-CRP (mg/L, $\bar{x}\pm s$)	13.58 ± 2.62	12.08 ± 2.67	0.009*	13.58 ± 2.62	11.57 ± 2.49	0.002*
PAGIn (pg/mL, $\bar{x}\pm s$)	619.20 ± 119.33	569.90 ± 113.14	0.046*	619.20 ± 119.33	555.64 ± 109.29	0.029*
TMAO (µmol/L, $\bar{x}\pm s$)	13.01 ± 2.19	12.01 ± 2.01	0.026*	13.01 ± 2.19	11.70 ± 1.78	0.011*
TBA (µmol/L, $\bar{x}\pm s$)	17.88 ± 6.02	15.04 ± 6.01	0.027*	17.88 ± 6.02	15.70 ± 6.31	0.150
GLP-1 (pmol/L, $\bar{x}\pm s$)	7.74 ± 2.07	8.63 ± 2.00	0.041*	7.74 ± 2.07	9.06 ± 2.11	0.012*

IL-6, interleukin-6; LPS, lipopolysaccharide; hs-CRP, high-sensitivity C-reactive protein; PAGIn, phenylacetyl glutamine; TMAO, trimethylamine-N-oxide; TBA, total bile acid; GLP-1, glucagons-like peptide-1; *p < 0.05, the difference was statistically significant.

### 3.3 Correlation Analysis between Risk Factors and Gensini Score

Spearman correlation analysis revealed a positive relationship between the Gensini score and the serum concentrations of IL-6 (r = 0.410, *p* < 0.001), hs-CRP (r = 0.317, *p* < 0.007) and TMAO (r = 0.311, *p* = 0.008). The detailed findings of this correlation are presented in Table 3.

**Table 3.** **Spearman correlation analysis between risk factors and Gensini score**.

	r value	*p* value
IL-6 (pg/mL)	0.410	<0.001*
LPS (pg/mL)	0.151	0.207
hs-CRP (mg/L)	0.317	0.007*
PAGIn (pg/mL)	0.099	0.412
TMAO (µmol/L)	0.311	0.008*
GLP-1 (pmol/L)	–0.183	0.127

*p < 0.05, the difference was statistically significant.

### 3.4 Multiple Linear Regression Analysis of Each Risk Factor and the Gensini Score

To further investigate the impact of various risk factors on the Gensini score, a multiple linear regression was performed on the statistically significant variables. The results indicated that IL-6 (b = 1.769, 95% confidence interval (CI): 0.256–3.282, *p* = 0.023) and TMAO (b = 10.735, 95% CI: 4.883–16.588, *p* ≤ 0.001) were key predictors of the Gensini score. The comprehensive results of this analysis are shown in Table 4.

**Table 4.** **Multiple linear regression analysis of each risk factor and Gensini score**.

	b value	β value	95% CI	*p* value
IL-6 (pg/mL)	1.769	0.261	0.256–3.282	0.023*
TMAO (µmol/L)	10.735	0.410	4.883–16.588	<0.001*

CI, confidence interval; *p < 0.05, the difference was statistically significant.

### 3.5 ROC Curves of IL-6 and TMAO for Predicting CAD

**Fig. 1.**
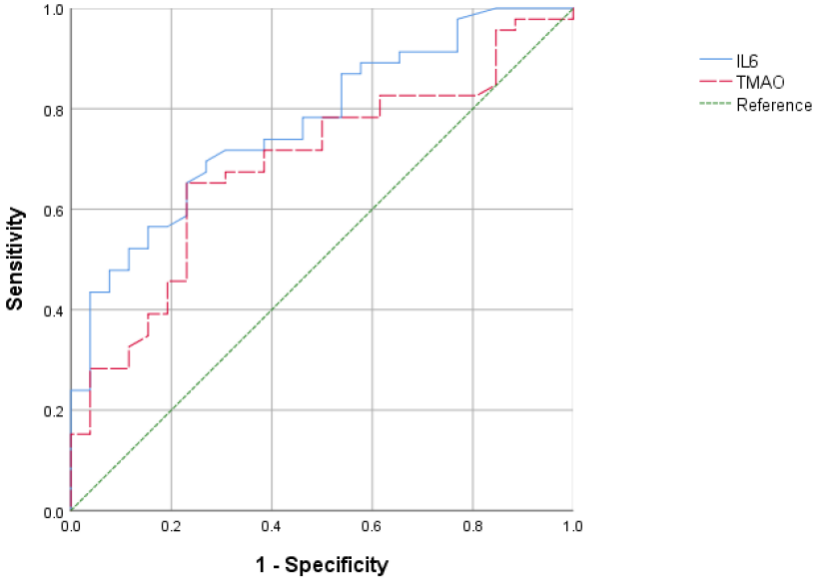
**ROC curves of IL-6 and TMAO for predicting CAD**. ROC, receiver operating characteristic.

The diagnostic efficacy of IL-6 and TMAO for CAD was evaluated using ROC curves. It was observed that the cut-off value of IL-6, set at 45.17, demonstrated the strongest diagnostic performance for CAD (sensitivity 69.6%, specificity 73.1%, area under the curve 0.770, 95% CI: 0.662–0.879, *p* < 0.001). Similarly, TMAO exhibited the highest diagnostic value for CAD (sensitivity 65.2%, specificity 76.9%, area under the curve 0.689, 95% CI: 0.564–0.814, *p* = 0.008) at a cut-off of 12.44. The corresponding ROC curves are displayed in Fig. 1.

### 3.6 Correlation Analysis between Risk Factors and CFR

A total of 18 randomly selected CAD patients underwent PET myocardial perfusion imaging to examine the relationship between risk factors and CFR. Spearman correlation analysis indicated a negatively correlation between TMAO and CFR (r = –0.593, *p* = 0.009). No significant correlations were found between CFR and other risk factors, including IL-6, LPS, hs-CRP, PAGIn, TMAO, and GLP-1 (*p* > 0.05). Since the sample size was limited, multiple regression analysis was not conducted. The results are summarized in Table 5.

**Table 5.** **Spearman correlation analysis between risk factors and CFR**.

	r value	*p* value
IL-6 (pg/mL)	–0.026	0.919
LPS (pg/mL)	–0.030	0.906
hs-CRP (mg/L)	–0.201	0.423
PAGIn (pg/mL)	–0.123	0.627
TMAO (µmol/L)	–0.593	0.009*
TBA (µmol/L)	–0.189	0.453
GLP-1 (pmol/L)	0.003	0.990

CFR, coronary flow reserve; *p < 0.05, the difference was statistically significant.

## 4. Discussion

As the global burden of CAD continues to rise annually, it has become a focal point of medical research and public health attention. Although significant advancements have been made in prevention strategies, pharmacological treatments, and interventional procedures, the incidence of CAD remains on an upward trajectory.

Recent evidence from numerous studies highlights the association between gut microbiota-derived metabolites and both the pathogenesis of AS and the prognosis of CAD. This investigation explored the relationship between the serum levels of IL-6, hs-CRP and TMAO, were positively correlation with the Gensini score, suggesting its potential role as a predictor of CAD severity. Furthermore, IL-6 was identified as an independent risk factor for the severity of CAD. The study also established that serum levels of IL-6 >45.17 pg/mL or TMAO >12.44 µmol/L had predictive significance for CAD diagnosis. Additionally, the serum concentration of TMAO showed a negative correlation with CFR, indicating its potential as a clinical biomarker for predicting CAD prognosis. Chronic inflammation, a critical factor in the onset and progression of AS, plays a vital role in triggering thrombosis and plaque rupture, thereby contributing to the poor prognosis of CAD [11]. This finding of this study indicate that IL-6 (r = 0.410, *p* < 0.001) and hs-CRP (r = 0.317, *p* = 0.007) exhibit a positive correlation with the severity of CAD, with IL-6 identified as a contributing risk factor for CAD severity (b = 1.769, 95% CI: 0.256–3.282, *p* = 0.023). It was observed that serum IL-6 levels exceeding 45.17 pg/mL possess predictive utility in diagnosis CAD. Research suggests that IL-6 mediates its biological functions through the IL-6R and gp130 signal transduction receptor complex on cardiomyocytes. This pathway is linked to poor CAD prognosis, facilitated by mechanisms such as platelet aggregation and the proliferation of coronary vascular smooth muscle cells, along others [12, 13]. IL-6 can also augment inflammation by increasing the production of acute inflammatory proteins including hs-CRP in the liver. Yang *et al*. [14] showed that hsCRP increase the risk of CAD. Similarly, Cheng *et al*. [15] demonstrated a positive relationship between the Gensini score, which quantifies the extent of coronary artery stenosis, and hs-CRP. These conclusions align with the outcomes of this investigation, which further revealed that hs-CRP acts as an independent risk factor for poor CAD prognosis.

LPS, a fundamental constituent of the Gram-negative bacteria cell wall, plays a crucial role in bacterial structure and function. Once the intestinal flora is altered, LPS is released by the bacterial membrane, which destroys the intestinal vascular barrier and leads to the absorption of LPS into the blood circulation, resulting in an increased systemic inflammatory response leading to sepsis. Serum analyses revealed that levels of LPS were considerably elevated in the CAD group compared to the non-CAD group. LPS can directly or indirectly induce the adhesion reaction between platelets and the vessel wall [16]. LPS can promote the production of foam cells through Toll-like receptor 4, CD36 and CD204, leading to plaque instability and eventually lead to AS.

PAG, a metabolite resulting from intestinal flora transformation, has been linked to CAD. Studies revealed that serum PAGIn concentrations in CAD patients were notably higher than those in non-CAD patients. Fang *et al*. [17] identified an elevation in blood PAGln levels in CAD patients with in-stent restenosis. Furthermore, Stanley Hazen’s team established a connection between PAGIn and cardiovascular disease, as well as adverse cardiovascular events, in a cohort of 4000 participants. Research on whole blood and isolated platelets from animal models with arterial injury revealed that gut microbiota-derived PAGln metabolites could promote platelet activation-related phenotypes and enhance thrombosis [18].

TMAO in human body is mainly derived from choline, L-carnitine, betaine, phosphatidylcholine, and lecithin, in red meat, egg yolk, fish, animal liver, soybean and other foods. TMAO is produced through the action of intestinal microorganisms, and TMAO is finally formed in the liver [19, 20]. The analysis revealed a significant positive correlatation between TMAO and the severity of CAD (r = 0.311, *p* = 0.008), identifying TMAO as an independent determinant of CAD severity (b = 10.735, 95% CI: 4.883–16.588, *p* < 0.001). Notably, when serum TMAO levels exceeded 12.44 µmol/L, its diagnostic value for CAD became apparent. Additionally, a negative relationship was observed between TMAO and the prognosis of CAD (r = –0.593, *p* = 0.009). Existing research highlights that gut microbiota influences serum TMAO levels, with evidence linking

TMAO to atherosclerotic plaque burden in healthy individuals. This association is independent of both renal function and dietary intake [21]. Multiple studies have demonstrated that TMAO serves as an independent predictor of mortality risk in CAD patients [22], and it acts as a concentration-dependent risk factor for CAD [23]. In a longitudinal study tracking 2235 individuals with stable CAD over a 5 years period, it was found that elevated TMAO levels significantly heightened the risk of cardiovascular death [24]. Research conducted by Waleed *et al*. [25] involving 4039 participants, further established that TMAO was independently linked to coronary atherosclerotic burden. Consistent with these findings, the present study also confirmed that TMAO is associated with both the severity of coronary artery lesions and the prognosis of coronary artery disease.

TBA is a key intermediate in the human body’s lipid metabolism pathway [26]. Bile acids affect lipid metabolism, glucose metabolism, energy metabolism, blood pressure regulation, and the inflammatory response by regulating the 15. farnesoid X receptor (FXR) and the Takeda G-protein-coupled receptor 5 (TGR5). The findings of this study revealed the serum in CAD higher than those in non-CAD patients. Clinical research indicates that the serum TBA concentrations in patients with essential hypertension are higher than those observed in healthy individuals, with a positive correlation observed between TBA concentration and the classification of hypertension. Studies have also shown that FXR agonist and taurocholate can reduce postprandial blood lipids in mice, and bile acids can promote coronary atherosclerosis by inhibiting FXR receptors [27].

GLP-1 is an incretin hormone rapidly secreted by intestinal endocrine L cells after meals. Studies have shown that the imbalance of intestinal flora can lead to the reduction of GLP-1 secretion. The results suggested that serum GLP-1 in CAD patient were lower than those in non-CAD, which is possible to be a protective factor. Helmstädter *et al*. [28] showed that GLP-1 and its analogues can reduce blood pressure and play a role in protecting endothelial function. GLP-1 can also play an anti-inflammatory role, regulate oxidative stress, improve energy metabolism, and ultimately play a protective role in cardiovascular disease.

In conclusion, derivatives originating from gut microbiota, such as IL-6, LPS, hs-CRP, PAGIn, TMAO and GLP-1, have a notable connection to the severity of CAD. Among these, IL-6, TMAO, and hs-CRP serve as independent risk indicators for the progression of CAD. When the serum cincentrations of IL-6 exceed 45.17 pg/mL or TMAO surpass 12.44 µmol/L, they hold predictive significance for CAD diagnosis. Additionally, TMAO is linked to the prognosis of CAD. The finding highlights that serum levels of IL-6, LPS, hs-CRP, PAGIn, TMAO and GLP-1 are markedly elevated in patients with a high risk of AS, providing a potential basis for patient risk stratification. Strategies involving probiotics and fecal transplantation are emerging as promising avenues for the prevention and mitigation of CAD. For instance, research indicates that probiotics and fecal transplantation may offer cardiovascular protection by lowering blood pressure [29, 30].

This study has several limitations. It is a single-center study and the sample size is small, which may affect the statistical power. The study sample was drawn from patients with suspected CAD who underwent coronary arteriography in the department of cardiology, rather than from a healthy population, which may have led to selection bias, although we used PSM to reduce this. In this study, only the serum levels of derivatives of gut microbiota was detected, and the lack of genetic detection was insufficient.

## 5. Conclusions

Serum biomarkers, including IL-6, LPS, hs-CRP, PAGIn, TMAO and GLP-1 can serve as clinical indicators for predicting the severity of CAD. Among them, IL-6, TMAO and hs-CRP are identified as independent risk factors for CAD severity. Elevated levels of IL-6 and TMAO possess predictive value for CAD diagnosis. Additionally, serum TMAO levels can act as a clinical marker to forecast the prognosis of CAD.

## Availability of Data and Materials

The datasets used and/or analyzed during the current study are available from the corresponding author on reasonable request.
